# Acu-TENS and Postexercise Expiratory Flow Volume in Healthy Subjects

**DOI:** 10.1155/2011/726510

**Published:** 2011-02-06

**Authors:** Shirley P. C. Ngai, Alice Y. M. Jones, Christina W. Y. Hui-Chan

**Affiliations:** ^1^Department of Rehabilitation Sciences, The Hong Kong Polytechnic University, Hung Hom, Kowloon, Hong Kong; ^2^Department of Physical Therapy, University of Illinois at Chicago, Chicago, IL 60612-7249, USA

## Abstract

Transcutaneous Electrical Nerve Stimulation over acupoints (Acu-TENS) facilitates recovery of resting heart rate after treadmill exercise in healthy subjects. Its effect on postexercise respiratory indices has not been reported. This study investigates the effect of Acu-TENS on forced expiratory volume in 1 second (FEV1) and forced vital capacity (FVC) in healthy subjects after a submaximal exercise. Eleven male subjects were invited to the laboratory twice, two weeks apart, to receive in random order either Acu-TENS or Placebo-TENS (no electrical output from the TENS unit) over bilateral Lieque (LU7) and Dingchuan (EX-B1) for 45 minutes, before undergoing exercise following the Bruce protocol. Exercise duration, rate of perceived exertion (RPE), and peak heart rate (PHR) were recorded. Between-group FEV1 and FVC, before, immediately after, at 15, 30, and 45minutes postexercise, were compared. While no between-group differences in PHR, RPE, and FVC were found, Acu-TENS was associated with a longer exercise duration (0.9 min (*P* = .026)) and a higher percentage increase in FEV1 at 15 and 45 minutes postexercise (3.3 ± 3.7% (*P* = .013) and 5.1 ± 7.5% (*P* = .047), resp.) compared to Placebo-TENS. We concluded that Acu-TENS was associated with a higher postexercise FEV1 and a prolongation of submaximal exercise.

## 1. Introduction

Aerobic training improves the body's ability to meet increased ventilatory demands during exercise, and ventilatory endurance contributes to improved exercise performance [1]. Exercise can increase airway resistance in subjects with reactive airways [2] which consequently leads to increased work of breathing and induces a sensation of shortness of breath, thereby limiting exercise performance and training intensity [1]. 

Within the concepts of Traditional Chinese Medicine (TCM), maintenance of health and normalization of body function stems from a balance of “yin” and “yang” and free flow of energy (Qi) along various meridians in the body [3]. Previous studies have suggested that acupuncture, a technique which facilitates free flow of Qi, can alleviate dyspnoeic symptoms [4, 5], reduce postexercise bronchoconstriction [6], and improve exercise capacity [4].

Acupuncture, though effective, is invasive and may be associated with complications such as pneumothorax, inadvertent puncture of internal organs, infection, and vasovagal reaction [7, 8]. Transcutaneous Electrical Nerve Stimulation (TENS), on the other hand, is a noninvasive modality, and has been widely used in clinical practice for analgesia [9]. Wang and coworkers [10] demonstrated that TENS triggered the release of frequency-dependent opioid receptors in the same way as acupuncture. Levin and Hui-Chan [11] showed that TENS excited large diameter afferents (A*α*, A*β*) in humans, similar to those excited by electro-acupuncture in animals [12]. Application of TENS over an acupuncture point (Acu-TENS) has been shown to facilitate the return to resting heart rate after an intensive treadmill exercise in healthy subjects [13]. Its effect on respiratory parameters after exercise in healthy subjects has not been reported. This study aims to investigate the effect of Acu-TENS on forced expiratory volume in one second (FEV_1_), a test of pulmonary function used to assess airway resistance, during sub-maximal treadmill exercise in healthy subjects.

## 2. Materials and Methods

Ethics approval was obtained from the departmental research committee of the involved university. Male subjects, aged 18 or above, with no known history of respiratory, cardiovascular, musculo-skeletal, neurological, or endocrine disorders, were invited to participate in the study. Male subjects only were recruited for the convenience of ECG monitoring during exercise. Study details were explained, and written consent was obtained from each subject prior to data collection. All subjects were asked to complete a questionnaire confirming an absence of any of the aforementioned disorders or upper respiratory tract infection during the 2 weeks prior to the commencement of the study.

### 2.1. Experimental Procedures (Figure 1)

This study adopted a single-blinded, randomized, crossover design. Male subjects (through “snow ball” invitation) with good general health were invited to visit the university laboratory twice, 2 weeks apart, as a washout period. They were asked to refrain from coffee, tea, and chocolate on the day of the visit [14]. Upon arrival at the university laboratory, subjects rested in a sitting position for 30 minutes to establish a steady state cardiopulmonary system. Baseline resting heart rate was monitored (S610; Polar; Finland). FEV_1_ and forced vital capacity (FVC) were measured according to the American Thoracic Society [15] guidelines using a spirometer (Pony, Cosmed, Italy) calibrated with a 3 L syringe prior to data collection. 

At the first visit, subjects were asked to draw from an opaque sealed envelope an allocation number indicating either Acu-TENS or Placebo-TENS as the first intervention, with the opposite intervention applied at their subsequent visit a fortnight later.

**Figure 2:**
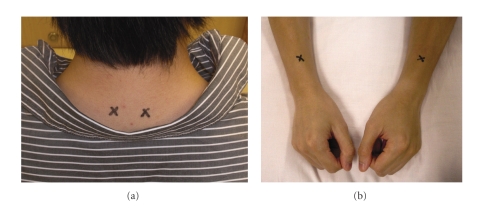
Electrode placement over (a) Dingchuan (EX-B1), (b) Lieque (LU 7).

1. Acu-TENS: subjects received TENS over the acupuncture points—Dingchuan (EX-B1) and Lieque (LU-7) for 45 minutes prior to exercise. Dingchuan is located 0.5 cun lateral to the lower border of 7th cervical vertebra (C7). Lieque is located 1.5 cun above the radial styloid process between the tendons of brachioradialis and abductor pollicis longus (Figures 2(a) and 2(b)). A “cun” is an ancient Chinese measurement unit used to locate an acupuncture point and is the distance between the medial ends of the creases of the middle and distal interphalangeal joints of the middle finger. This finger-cun method was used for location of the acupuncture points, because our previous work has demonstrated that stimulation of the acupuncture points located by this method was effective [13, 16, 17]. The intensity of stimulation was increased until it produced a painful stimulus and then reduced until tolerable.
2. Placebo-TENS: it was identical to the Acu-TENS group except that there was no electrical current delivered by the machine to the subject. To enhance Placebo credibility, a display of an intensity output light and a countdown of intervention timer were activated.

Skin area over the selected acupuncture points was cleaned with an alcohol swab. Four electrodes of 20 × 20 mm^2^, filmed with aqueous gel, were then placed over the points and attached to a dual channel portable TENS unit (ITO 320; ITO Co. Ltd; Japan). Stimulation frequency was adjusted to 2 Hz, with a pulse width of 200 *μ*s. Prior to data collection, voltage, pulse width, and frequency output from the TENS unit were verified as being consistent and accurate by means of a 100 MHz Oscilloscope (MSO6014A; Agilent Technologies; USA). Subjects were informed that, depending on the frequency used, they might or might not feel electrical stimulation during the intervention. 

Subjects were then asked to perform treadmill exercise adopting the Bruce protocol which was terminated when maximal exercise intensity was reached [18] or when the subject felt too tired or short of breath to continue the exercise. Lung function parameters and heart rate were measured in a sitting posture, immediately after, and at 15, 30, and 45 minutes after exercise. Exercise duration, rate of perceived exertion (RPE), and peak heart rate (PHR) were recorded at the termination of exercise. Room temperature and relative humidity in the laboratory were maintained at constant levels during both visits.

## 3. Statistical Analysis

Demographic and baseline data including age, height, weight, and body mass index (BMI) were recorded. Pre-exercise heart rate, FEV_1_, FVC, percentage of predicted FEV_1_ and FVC, exercise duration, PHR, percentage of maximal heart rate, and RPE reached at the end of exercise were recorded at the 2 visits and were compared by paired *t*-test. Percentage changes of FEV_1_ and FVC at each time point (i.e., immediately after, and at 15, 30, and 45 minutes postexercise) were compared between the Acu-TENS and Placebo-TENS groups using repeated measures ANOVA followed by post hoc analysis. All data were analysed using the statistical software (SPSS for Windows version 16; SPSS, Chicago, IL). Type I error level was set at *α* = 0.05.

## 4. Results

**Figure 3:**
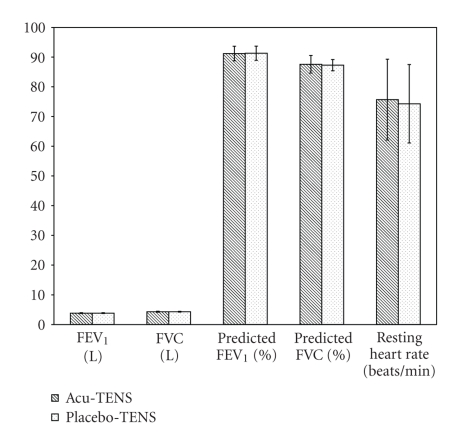
Baseline physiological parameters recorded before interventions.

Eleven male subjects participated in the study. Their mean age (±SEM) and BMI were 25.0 ± 1.3 years and 23.4 ± 1.4 kgm^−2^, respectively. The mean cun was 2.1 ± 0.0 cm. Nine subjects undertook daily exercise and participated in regular soccer matches. Ten were nonsmokers while one was an exsmoker who had stopped smoking for 2 years. All subjects had basal FEV_1_ and FVC which were more than 85% of the predicted value and an FEV_1_/FVC ratio >75%. There were no differences in preintervention baseline variables between the two visits (*P* > .05) (Figure 3). No adverse effect was reported.

**Figure 4:**
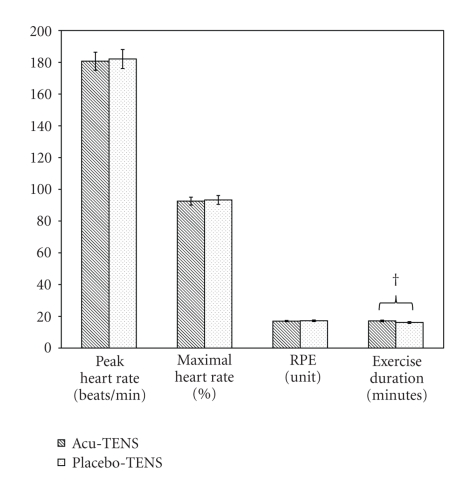
Variables recorded immediately postexercise. RPE: rate of perceived exertion; † denotes *P* < .05.

The findings showed that the percentage of maximal heart rate reached was 92.6 ± 2.5% with Acu-TENS and 93.3 ± 2.8% with Placebo-TENS at termination of exercise (Table 1, Figure 4). Exercise duration was, however, longer with Acu-TENS when compared with Placebo-TENS intervention by 0.9 min (*P* = .026), but there were no differences in PHR and RPE between the two interventions (Table 1, Figure 4).

**Figure 5:**
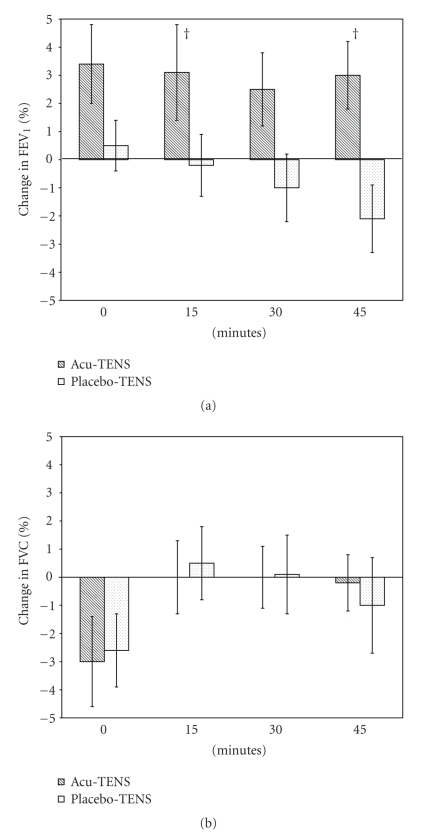
Percentage change in postexercise (a) Forced Expiratory Volume in 1 second (FEV_1_) and (b) Forced Vital Capacity (FVC). † denotes *P* < .05.

After exercise, there was an increase in FEV_1_ of 2.5% to 3.4%, over all time points in the Acu-TENS group. Placebo-TENS group showed a slight increase in FEV_1_ immediately after exercise but then a decrease of FEV_1_ 45 minutes postexercise. Between-group differences in the percentage change of FEV_1_ were significant at 15 minutes (3.3 ± 1.1% (*P* = .013)) and 45 minutes (5.1 ± 2.2% (*P* = .047)) (Table 1, Figure 5(a)). The change in FVC did not reach a statistically significant level (Table 1, Figure 5(b)).

## 5. Discussion

Our study demonstrated that subjects after Acu-TENS had an increase in FEV_1_ after exercise while a reduction in postexercise FEV_1_ was associated with Placebo-TENS. Such a difference was statistically significant at 15 and 45 minutes after exercise. Subjects who received Acu-TENS could exercise for a longer duration at near maximal exercise intensity (>90% of their predicted heart rate maximum) compared to when they received Placebo-TENS treatment. 

Airway diameter is primarily influenced by parasympathetic nervous system fibres with sparse sympathetic innervations [19]. Parasympathetic control dominates at rest, but vagal tone is reduced or even abolished during exercise [20], thereby leading to relaxation of the airway smooth muscle and consequently, bronchodilation. This mechanism is a possible explanation for the initial increase in FEV_1_ observed immediately after exercise in our subjects, irrespective of the intervention. Respiratory data in our Placebo-TENS group were similar to those reported by de Bisschop and coworkers [21]. In their cohort of healthy subjects, a bronchodilation effect appeared during exercise but lasted only 90 seconds after exercise, followed by an immediate increase in airway resistance. It has been suggested that this postexercise bronchoconstrictor effect is mediated by the prompt restoration of vagal tone at exercise cessation [22]. 

Whether any immediate change in airway calibre occurs in healthy subjects, during and/or after exercise, has been a controversial issue [2]. Some researchers have reported no change in airway resistance during exercise [23–25], while others, a decrease [26, 27]. Variation in exercise intensity and duration among these studies may account for the inconsistent findings. In this current study, subjects receiving Acu-TENS demonstrated an increase in postexercise FEV_1_ which was maintained for 60 minutes. They were also able to exercise at sub-maximal exercise intensity for almost a minute longer than subjects in the Placebo-TENS group. 

Low-frequency, high-intensity TENS has been shown to trigger the release of endorphin [28–30]. Effect of endorphin on the respiratory centre has been reported to suppress exercise-induced hyperventilation [31–33]. Stimulation of opioid *μ*-receptors was shown to specifically augment beta-adrenoceptor-mediated bronchodilation in a canine model [34]. Sluka and Walsh [35] conducted a series of animal studies which demonstrated that the type of opioid release induced by TENS is frequency dependent. Furthermore, low-frequency, high-intensity TENS was reported to be effective in triggering *μ*- and *δ*-opioid receptors [30]. Stimulation of these opioid receptors can affect respiratory control leading to a decrease in respiratory rate [33, 36, 37]. Our recent work has demonstrated that 45 minutes of Acu-TENS improved FEV_1_ and diminished dyspnoea sensation in patients with chronic obstructive pulmonary disease, and these effects were associated with an increase in blood endorphin levels [16, 17]. Furthermore, the improvement in FEV_1_ was only demonstrable in patients in whom the electrical stimulation was applied over acupuncture points and not in the patient cohort treated with non-acupoint stimulation (electrical stimulation over the patella). This suggests that the effect of Acu-TENS on FEV_1_ is point specific [16].

**Figure 6:**
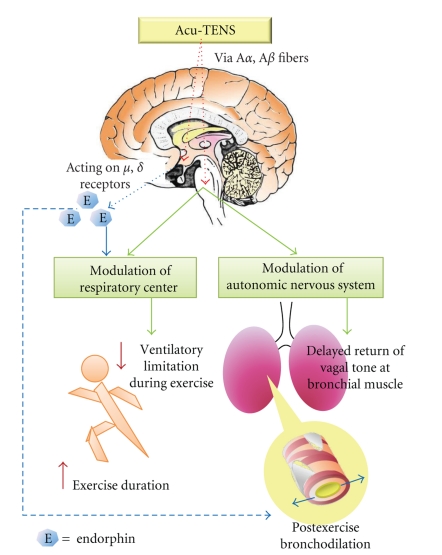
A hypothetical diagram illustrating a mechanism of effect on exercise duration and postexercise airway response of Acu-TENS.

Muscle contractions resulting from low-frequency, high-intensity TENS will excite large diameter afferents [11, 12], stimulation of which has been shown to release endogenous opioids [11, 30]. Neural output from the respiratory centre is influenced by exercise-induced opioid release [32]. It is postulated that Acu-TENS applied prior to exercise will excite large diameter afferents both directly and indirectly though muscle contractions, which trigger the release of endogenous opioids, which then continues during exercise. The duration of endogenous opioid effect can last more than 1 hour [38], and this might explain the sustained increase in FEV_1_ after exercise. Endogenous opioid release during exercise may attenuate ventilatory limitation during exercise either centrally at the respiratory centre and/or peripherally by directly lowering airway resistance, thereby prolonging exercise duration (Figure 6). 

An increase in sympathetic tone in bronchial muscle is associated with bronchodilation, while an increase in parasympathetic tone induces bronchoconstriction [39]. Stimulation of certain acupuncture points such as NeiGuan (PC6) is believed to induce an increase in parasympathetic tone, thereby affecting the heart rate [40]. The improvement in FEV_1_ in our subject cohort after Acu-TENS could be associated with changes in autonomic nervous system activity. Investigation of the effects of Acu-TENS on the autonomic nervous system was however beyond the scope of this current study. 

Table 1 showed a 3.3% and 5.1% between-group difference in FEV_1_ at 15 and 45 minutes, respectively, after exercise. This change corresponds to an expired volume of less than 200 mL. Such a degree of change is not considered “clinically significant” [41]. However, as Gotshall [2] pointed out, in normal individuals, the airway calibres are in a near-maximal or maximally dilated state, and so further dilation upon exercise is likely to be of small magnitude. Kagawa and Kerr [27] and Warren and coworkers [42], however, showed that exercise induced a powerful bronchodilating effect in healthy individuals, but the airways of their subjects were “preconstricted” by oral propanolol prior to the exercise. Although the magnitude of improvement in FEV_1_ in our subjects with normal airway diameter appeared to be small, the relative change induced in subjects with reactive or narrowed airways may be of greater clinical significance. 

In summary, this study demonstrated that the application of Acu-TENS produced a slight but significant increase in postexercise FEV_1_ together with a capacity to prolong sub-maximal exercise duration. Our hypothesis that the sustained increase in postexercise FEV_1_ could be a result of endogenous opioid neurohumoral modulation and/or a consequence of altered autonomic nervous system activity requires further investigation.

## 6. Conclusion

Contrary to Placebo-TENS, our findings showed a statistically significant enhancement of postexercise FEV_1_ and prolongation of sub-maximal exercise duration after administration of Acu-TENS in subjects with normal health. In other words, Acu-TENS, a noninvasive modality, is able to attenuate the drop in FEV_1_ and enhance the respiratory response to exercise. Our study suggests that Acu-TENS has the potential to improve the postexercise FEV_1_ and may have a role in extending the duration of exercise training.

## Figures and Tables

**Figure 1:**
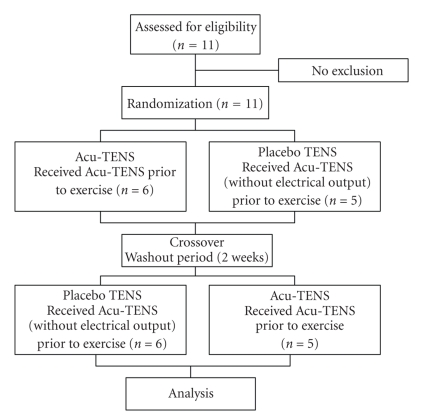
Flow diagram of the study procedure.

**Table 1:** Variables recorded at different postexercise time points.

	Acu-TENS *n* = 11	Placebo-TENS *n* = 11	Mean difference Acu-TENS − Placebo-TENS (95%CI)	*P* value
Peak heart rate (beats per min)	180.7 ± 5.7	182.1 ± 6.0	−1.4 (−7.1 to 4.3)	.606
Heart Rate, % maximum	92.6 ± 2.5	93.3 ± 2.8	−0.7 (−3.7 to 2.2)	.601
Rate of perceived exertion	17.0 ± 0.4	17.2 ± 0.5	−0.2 (−0.6 to 0.2)	.341
Exercise duration, min	17.1 ± 0.5	16.1 ± 0.6	0.9 (0.1 to 1.7)	.026^†^
Δ FEV_1_ postexercise, %				
0 min	3.4 ± 1.4	0.5 ± 0.9	2.9 (−0.6 to 6.4)	.098
15 min	3.1 ± 1.7	−0.2 ± 1.1	3.3 ( 0.9 to 5.8)	.013^†^
30 min	2.5 ± 1.3	−1.0 ± 1.2	3.5 (−1.3 to 8.3)	.135
45 min	3.0 ± 1.2	−2.1 ± 1.2	5.1 (0.1 to 10.1)	.047^†^
Δ FVC postexercise, %				
0 min	−3.0 ± 1.6	−2.6 ± 1.3	−0.4 (−3.5 to 2.7)	.784
15 min	0.0 ± 1.3	0.5 ± 1.3	−0.5 (−3.7 to 2.8)	.763
30 min	0.0 ± 1.1	0.1 ± 1.4	−0.1 (−3.5 to 3.4)	.974
45 min	−0.2 ± 1.0	−1.0 ± 1.7	0.8 (−3.6 to 5.2)	.705

Data are mean ± SEM unless otherwise indicated. † denotes *P* < .05; FEV_1_: forced expiratory volume in one second, FVC: forced vital capacity, HR: heart rate; Δ denotes percentage change: (postexercise data − pre-exercise data)/pre-exercise data × 100%.
